# Understanding Gene Sequence Variation in the Context of Transcription Regulation in Yeast

**DOI:** 10.1371/journal.pgen.1000800

**Published:** 2010-01-08

**Authors:** Irit Gat-Viks, Renana Meller, Martin Kupiec, Ron Shamir

**Affiliations:** 1 Broad Institute of Harvard and Massachusetts Institute of Technology, Cambridge, Massachusetts, United States of America; 2 School of Computer Science, Tel Aviv University, Tel Aviv, Israel; 3 Department of Molecular Microbiology and Biotechnology, Tel Aviv University, Tel Aviv, Israel; North Carolina State University, United States of America

## Abstract

DNA sequence polymorphism in a regulatory protein can have a widespread transcriptional effect. Here we present a computational approach for analyzing modules of genes with a common regulation that are affected by specific DNA polymorphisms. We identify such regulatory-linkage modules by integrating genotypic and expression data for individuals in a segregating population with complementary expression data of strains mutated in a variety of regulatory proteins. Our procedure searches simultaneously for groups of co-expressed genes, for their common underlying linkage interval, and for their shared regulatory proteins. We applied the method to a cross between laboratory and wild strains of *S. cerevisiae*, demonstrating its ability to correctly suggest modules and to outperform extant approaches. Our results suggest that middle sporulation genes are under the control of polymorphism in the sporulation-specific tertiary complex Sum1p/Rfm1p/Hst1p. In another example, our analysis reveals novel inter-relations between Swi3 and two mitochondrial inner membrane proteins underlying variation in a module of aerobic cellular respiration genes. Overall, our findings demonstrate that this approach provides a useful framework for the systematic mapping of quantitative trait loci and their role in gene expression variation.

## Author Summary

High-throughput genotypic and expression data for individuals in a segregating population can provide important information regarding causal regulatory events. However, it has proven difficult to predict these regulatory relations, largely because of statistical power limitations. The use of additional available resources may increase the accuracy of predictions and suggest possible mechanisms through which the target genes are regulated. In this study, we combine genotypic and expression data across the segregating population with complementary regulatory information to identify modules of genes that are jointly affected by changes in activity of regulatory proteins, as well as by genotypic changes. We develop a novel approach called *ReL analysis*, which automatically learns such modules. A unique feature of our approach is that all three components of the module—the genes, the underlying polymorphism, and the regulatory proteins—are predicted simultaneously. The integrated analysis makes it possible to capture weaker linkage signals and suggests possible mechanisms underlying expression changes. We demonstrate the power of the method on data from yeast segregants, by identifying the roles of new as well as known polymorphisms.

## Introduction

DNA sequence polymorphisms that alter the activity of regulatory proteins can have considerable effect on gene expression [1]–[3]. With the advent of microarray and other genotyping technologies, it is now possible to examine the genome-wide effects of naturally occurring DNA sequence polymorphism on gene expression variation in segregating populations. For example, genotyping and expression data have been measured for 112 segregants obtained from a cross between the laboratory (BY) and wild (RM) strains of *S. cerevisiae* [1] and for 111 BXD mouse strain segregants [3].

Linkage analysis is commonly employed to identify DNA sequence polymorphism underlying gene expression phenotypes [1], [3]–[14]: the gene expression levels are treated as quantitative traits and the underlying DNA polymorphisms are called expression quantitative trait loci (eQTLs). Although standard linkage analysis successfully identifies eQTLs when applied to relatively small datasets, its utility in high-throughput eQTL analysis is limited due to the increased amount of background noise. To tackle this problem, a variety of methods take advantage of the modularity of biological systems and identify sequence polymorphisms that underlie an entire group of genes rather than single gene expression traits [4]–[6],[8],[10],[12]. Alternatively, a number of integrative approaches combine several data sources, including promoter binding data and sequence information, to improve the accuracy of eQTL identification [3],[14]. Several advanced methods capture not only sequence polymorphisms, but also the regulatory proteins underlying the expression changes. In those methods, the regulatory proteins are inferred concurrently with the linkage analysis, based on the approximation of regulatory protein activities by their mRNA expression level (e.g., [4],[12]).

**Figure 1:**
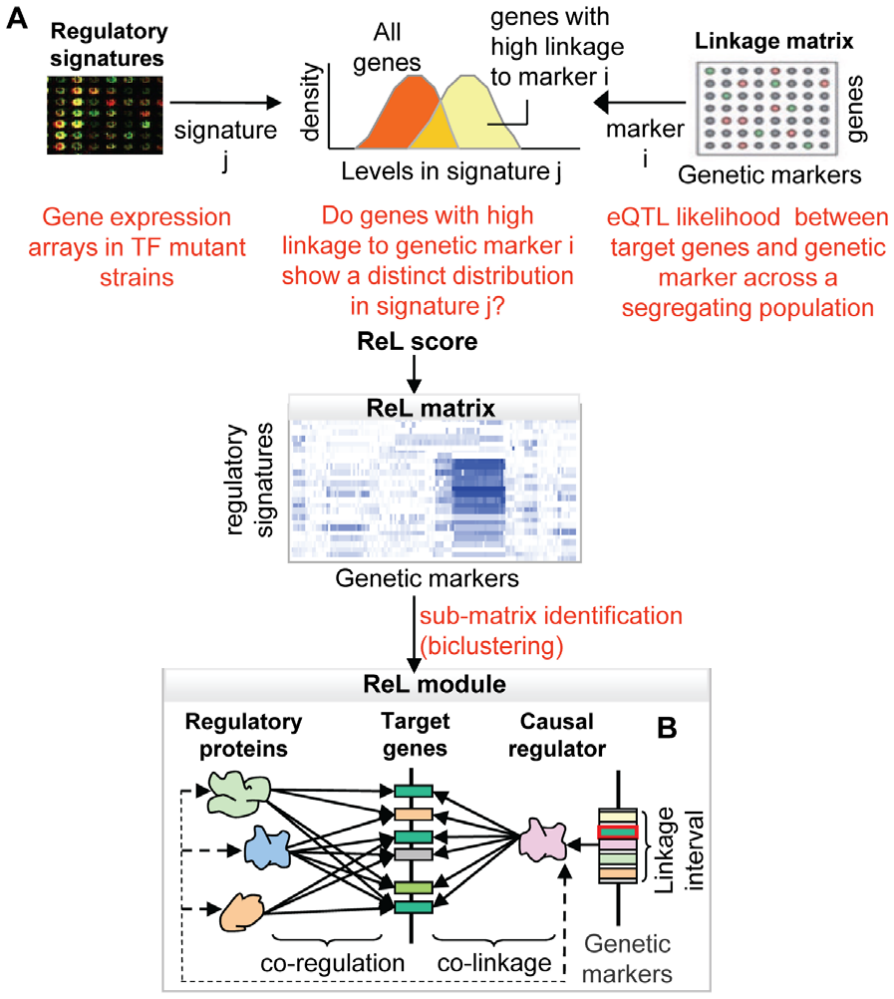
Overview of the ReL analysis procedure. (A) The “linkage matrix” contains eQTL likelihood scores between particular genetic markers (columns) and target genes (rows). The compendium of regulatory signatures consists of gene expression arrays from strains mutated in a variety of regulatory proteins. First, we aim to detect groups of genes that are co-expressed in one signature and co-linked to a specific genetic marker. Given a regulatory signature j and genetic marker i, we ask to what extent genes with high linkage to i manifest a distinct distribution in signature j. The difference between the distributions is estimated, producing a P-value called “ReL score.” The “ReL matrix” represents these scores across all regulatory signatures (columns) and genetic markers (rows). Blue/white indicates significant/non-significant ReL scores. Next, we aim to detect a group of genes that are co-expressed in a few signatures and co-linked to a common linkage interval. To that end, we look for high-scoring sub-matrices in the ReL matrix, referred as “ReL modules.” (B) Each ReL module represents a triplet: (i) a range of genetic markers (linkage interval) that contains a genetic causal regulator, (ii) a set of regulatory signatures matching particular regulatory proteins, and (iii) a group of target genes that are co-regulated by the module's regulatory proteins and co-linked to the module's causal regulator.

In this study we devise a new method for characterizing the transcriptional response to DNA sequence variation. Called Regulatory-Linkage (ReL) analysis, it captures groups of genes together with their underlying DNA polymorphisms and their common regulatory mechanisms. The method (Figure 1A) takes as input genotyping and expression data for individuals in the segregating population, as well as a compendium of high-throughput transcription regulatory signatures. These regulatory signatures are gene expression profiles (selected from the literature) of strains mutated in particular regulatory proteins, such as transcription factors and chromatin modifiers. Our method produces a set of ‘ReL modules’, each consisting of a triplet: a small set of regulatory proteins, a group of target genes, and a genetic linkage interval. The target genes are jointly linked to the interval and share a common transcriptional control by the regulatory proteins. We say that the module's target genes are co-regulated by the module's regulatory proteins and are co-linked to the modules' linkage interval (Figure 1B).

The novelty of the current approach is twofold. All three components of the ReL modules – the groups of target genes, the underlying polymorphism and the regulatory proteins – are predicted simultaneously. Extant methods predict only two of the components simultaneously and add the third one in a separate pre- or post-processing step. Moreover, we integrate high-throughput gene expression data consisting of perturbations in a large variety of transcription factors. This integrated approach has several important benefits: First, the additional regulatory information makes it possible to capture weaker linkage signals. Second, the analysis focuses on groups of target genes that have a common regulatory protein and therefore avoids groups of genes that happen by chance to be co-linked to the same genomic interval. Third, the approach infers regulatory relations based on perturbations in a variety of regulatory proteins, thereby avoiding the approximation of protein activities by mRNA expression levels. Previous studies relied on this rough approximation to infer regulatory proteins concurrently with DNA polymorphisms (e.g., [4],[12]). Finally, the predicted regulatory proteins may suggest possible mechanisms through which genetic polymorphisms affect their target genes, providing initial interpretations of the ReL modules as part of the analysis.

## Results

Our analysis takes, as input, genotypic and expression data for a set of 112 individuals in a yeast wild-type segregating population. We organize these data as a linkage matrix, which presents the linkage (an eQTL likelihood score) between the expression level of each gene and each genetic marker (Figure 1A; see Methods). In addition, our procedure utilizes a compendium of ‘regulatory signatures’ that includes gene expression profiles from 283 different strains mutated in a variety of regulatory proteins [15]–[16]. In the following analysis, linkage relations are evaluated based on the linkage matrix, whereas regulatory relations are assessed by preferential over- or under-expression of target gene groups across regulatory signatures.

We aim to identify triplets of (i) target genes, (ii) linkage interval, and (iii) regulatory signatures, where the target genes are jointly linked to the linkage interval and co-expressed in the regulatory signature. The naïve approach of finding high-scoring triplets by evaluating all possible combinations is computationally infeasible even for relatively small datasets. To tackle this problem, our method proceeds heuristically in two stages. In the first stage, we organize the input as a higher order ‘ReL matrix’ across all genetic markers and regulatory signatures (Figure 1A). Each entry in the matrix indicates whether genes that are strongly linked to a particular marker are also over- or under-expressed in a particular regulatory signature. This statistical measure, referred to as ReL score, is calculated as follows: For each genetic marker, we partitioned the genes into two sets: genes with high linkage to the genetic marker and the rest of the genes. Given the regulatory signature, the ReL score measures the difference in the gene expression distribution between these two sets (see Methods).

We now use the observation that when a group of genes is co-regulated by several regulatory proteins and is jointly linked to the same linkage interval, the corresponding ReL sub-matrix will attain high scores. In accordance, the second analysis stage (Figure 1A) applies a biclustering algorithm on the ReL matrix to search for sub-matrices whose average scores are higher than randomly expected. In this work, we assume a single linkage interval underlying each sub-matrix. Accordingly, the ISA biclustering algorithm [17] was adapted to choose a single range of genetic markers (Methods).

The biclustering output is a set of sub-matrices, each scored by its average ReL scores, and specifies a set of regulatory signatures and a single linkage interval. For each high-scoring sub-matrix, referred to as ReL module, we attached additional attributes: (i) A set of regulatory proteins – the proteins that were mutated in the strains from which the module's regulatory signature was obtained. (ii) A group of target genes - genes that are both co-regulated by the module's regulatory proteins and co-linked to the module's linkage interval (see Methods). Since we focus only on trans-acting regulation, genes residing within or near the modules' linkage interval were excluded from the group of target genes. (iii) We hypothesize that the linkage interval contains a single gene that underlies the module's gene expression variation. We call this gene the causal regulator of the module. Among the genes within the linkage interval, we predict a plausible putative causal regulator (see Methods; Figure 1B).

In this analysis, we focus on the thirteen highest-scoring ReL modules (modules with ReL score >3). A comprehensive description of these modules is given in Table S1 and Table S2. Five additional modules were highly enriched in target genes residing in telomeric or subtelomeric regions of multiple chromosomes, and therefore were excluded from the analysis (Table S2; gene expression variation in telomeres has been discussed extensively elsewhere (e.g., [4])). Each of the identified ReL modules consists of at least 10 target genes. The modules comprise a total of 311 genetic markers, 82 different regulatory proteins, and 281 different target genes. Randomization analysis shows that the identified modules are highly unlikely to be generated at random (module size P-value<0.05, see Text S1 for details).

**Figure 2:**
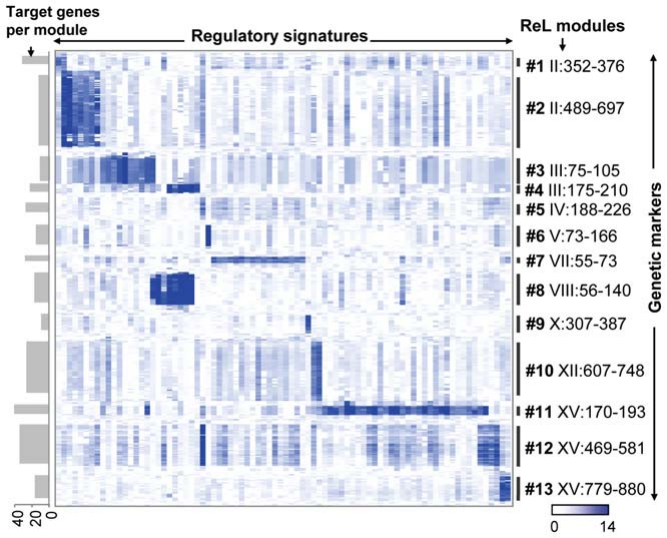
The ReL matrix. A matrix of genetic markers (rows) versus regulatory signatures (columns), where the colors indicate the ReL score. A blue (white) entry indicates significant (non-significant) ReL score. Shown are only columns and rows that are part of at least one ReL module as well as five additional flanking genetic markers on the boundaries of the module's linkage interval. Genetic markers are displayed according to their genomic order, and the modules' linkage intervals are shown as black vertical lines. Columns are grouped according to the biclustering solution whereas columns that are shared among multiple modules are assigned to one of their modules arbitrarily. The visualization highlights the existence of high intensity sub-matrices (modules) within the ReL matrix. The number of target genes in each module is shown as gray bars on the left.

The identified ReL modules have no overlapping linkage intervals and only a few shared regulatory proteins: Eleven regulatory signatures are shared across two modules and no regulatory signature is shared across three or more modules. This is likely to be a consequence of our biclustering approach and the small number of modules. The little overlap allows us to organize the ReL matrix into a global map of ReL modules (Figure 2). The global map highlights the existence of ‘high intensity’ sub-matrices (modules). The map clearly shows that the high ReL scores within each module decrease drastically at the boundaries of its linkage interval and for regulatory signatures that are not part of the module.

Table 1 summarizes the ReL modules and their function. Modules are listed along with their key (best-scoring) regulatory protein, putative causal regulator, and the biological processes most enriched in the target genes (based on enrichment test; see Table S3). For example, the nucleobase biosynthesis module (module #6) predicts that uracil biosynthetic enzymes are linked to the causal regulator *URA3* and regulated by the transcription factor Ppr1. Indeed, Ppr1 is a known transcription regulator of uracil biosynthesis genes, and the RM parental strain carries a deletion of *URA3*, a gene encoding one of the uracil biosynthetic enzymes (see details below).

**Table 1:** Summary of ReL modules.

#Module	ReL score	Linkage interval	Regulatory signatures	Target genes	Primary biological process	Best-scoring regulatory protein	Putative causal regulator
1	3.5	II:352–376	2	32	ribosome biogenesis	Pib2	TRM7
2	5.5	II:489–697	8	13	cytokinesis	Ace2	AMN1
3	10.4	III:75–105	10	12	leucine biosynthesis	Leu3	LEU2
4	11.4	III:175–210	6	23	response to pheromone	Ste12	MATα1,2
5	3.2	IV:188–226	1	28	oxidative phosphorylation	Swi3	CRD1
6	11.6	V:73–166	1	16	nucleobase biosynthesis	Ppr1	URA3
7	3.6	VII:55–73	17	29	ribosome biogenesis	Stb3	TAN1
8	18.4	VIII:56–140	8	18	conjugation	Ste12	GPA1
9	6.3	X:307–387	1	10	zinc-dependent process	Tec1	ZAP1
10	4.0	XII:607–748	2	27	ergosterol metabolism	Reb1	HAP1
11	6.8	XV:170–193	31	41	energy reserve metabolism	Mga2	IRA2
12	4.9	XV:469–581	5	35	oxidative phosphorylation	Swi3	CAT5
13	6.4	XV:779–880	2	17	sporulation	Sum1, Hst1	RFM1

A list of all modules with ReL score >3. The columns in the table (left to right) are as follows: module number, the module's ReL score, linkage interval, number of regulatory signatures, number of target genes, the primary biological process of the module (Table S3), the best-scoring regulatory protein of the module, and the predicted causal regulator of the module (Text S2). A comprehensive description of these ReL modules is given in Table S1.

All thirteen modules are significantly associated with a biological process (Table 1; eleven significant enrichments based on the GO database and two additional enrichments based on SGD, see Table S3). These significant enrichments give further support to the inferred ReL modules. For example, they justify the division of linkage interval II:352–697kb into two neighboring modules, #1 and #2 (linkage intervals II:352–376kb and II:489–697kb, respectively), since each module is characterized by a different biological process (‘ribosome biogenesis’ and ‘cytokinesis’, respectively; Table S3). Module #1 consists of 32 target genes, including ten ribosome biogenesis genes and only one cytokinesis gene. In contrast, module #2 consists of thirteen target genes with seven cytokinesis genes and no ribosome biogenesis genes (Table S1).

Among the genes residing within the linkage interval, the putative causal regulators (Table 1) were identified based on three criteria: (i) genes sharing the same biological process as the target genes, (ii) genes that have a physical interaction with at least one of the module's regulatory proteins, or (iii) proteins having a preferential binding to the promoter of the target genes (see Methods and Text S2 for a comprehensive description of causal regulator identification). For example, we have two indications that the causal regulator *URA3* underlies gene expression variation in module #6. First, it takes part in the same biological process as the target genes (nucleobase biosynthesis), and second, it physically interacts with the module's regulatory protein Ppr1.

Out of the thirteen putative causal regulators, seven were previously confirmed (*LEU2*, *URA3*, *AMN1*, *MAT*, *GPA1*, *HAP1*, *IRA2*; [1], [6], [18]–[19]), thereby serving as positive controls. Two other putative causal regulators (*ZAP1* and *CAT5*) were proposed previously but have not been tested [4],[6]. Two previously confirmed eQTLs (*MKT1* and *FLO8* [1],[5]) are not included in our ReL modules. Four putative causal regulators, *RFM1*, *CRD1*, *TRM7* and *TAN1* (modules #1, #5, #7, and #13), have not been previously identified.

The ReL analysis predicts regulatory relations between the modules' regulatory proteins and target genes. To demonstrate the quality of these predictions, we present their agreement with known, well-established transcriptional relations. Out of six known relations, ReL detects five relations whereas compared methods detect zero and four relations (see Text S3 for details). Interestingly, the nucleobase biosynthesis system was detected only by the ReL analysis.

The nucleobase biosynthesis system (module #6; Table 1) shows the unique ability of ReL analysis to recover not only the causal regulators, but also the regulatory proteins. The module's causal regulator is *URA3*, the target genes consist of *URA1* and *URA4*, and the highest scoring regulatory protein is Ppr1. The module successfully captures the current biological knowledge about the uracil biosynthesis system. The RM parental strain carries a deletion of the *URA3* gene, which is known to be linked to several members of the uracil biosynthesis pathway [1]. De-novo uracil biosynthesis is catalyzed by seven biosynthetic enzymes (Ura2,3,4,5,6,7,10). Four biosynthetic enzymes (Ura1,3,4,10) are subject to transcription regulation via the transcriptional activator Ppr1, whose activity is negatively regulated by uracil production rate [20]. The predicted effect of *URA3* mutation on *URA1,4* is highly likely to be mediated by Ppr1 activity: in the absence of Ura3 (RM variant), uracil production is reduced, causing Ppr1 activation (through the negative feedback) and, consequently, a transcriptional up-regulation of the uracil biosynthetic genes.

Notably, although most extant methods detect the nucleobase biosynthesis module, our approach is unique in inferring Ppr1 as the regulatory protein of the module (Text S3). This difference is not surprising, as most extant methods estimate Ppr1 activity by its mRNA level, whereas the actual activity is governed by uracil production rate. Taken together, the nucleobase biosynthesis module highlights the advantage of ReL analysis in predicting regulatory proteins based on causal information, without estimating protein activities with mRNA levels.

**Figure 3:**
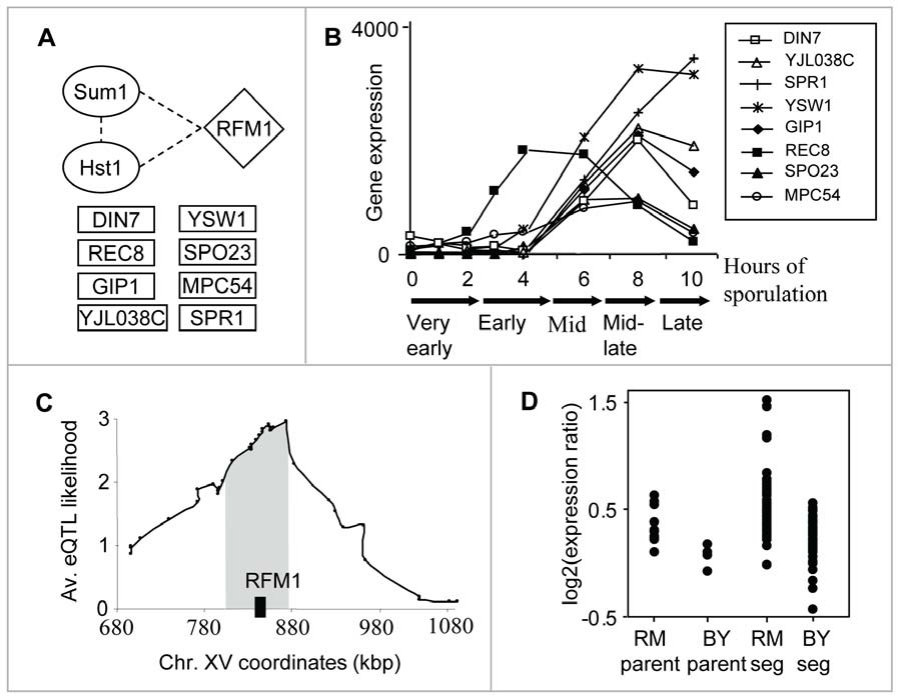
The sporulation module (#13). (A) The components: the modules' regulatory proteins (in ovals), its putative causal regulator (diamond-shaped), and eight meiosis-/sporulation-specific target genes (in rectangles). Protein–protein interactions between the components are indicated as dashed lines (Text S2). (B) Gene expression patterns (y-axis) of the eight meiosis-/sporulation-specific target genes through the sporulation time course ([30]; x-axis). The plot shows a temporal induction of the target genes during mid-sporulation. (C) The linkage plot. The eQTL likelihood (y-axis) is plotted for each genetic marker position along the module's linkage interval and its flanking regions (x-axis). Rfm1, a subunit of the Sum1p/Rfm1p/Hst1p complex that controls mid-sporulation genes, is located within the eQTL likelihood peak (black box). (D) Expression levels of parents and segregants for a marker in *SNR31*, which lies near *RFM1*. The plot shows average expression levels over the target genes for six replicates of each parent and for segregants that inherited the marker from BY and RM. The effect of the locus is in the same direction as the difference between the parents.

The sporulation module (module #13) shows our method's ability to reveal small modules. This module consists of only seventeen genes, eight of which encode meiosis- and sporulation-specific proteins (Figure 3A), linked to a locus on chromosome XV. Using previously reported mRNA expression patterns of all yeast genes through the sporulation time course, we found that these target genes are induced during mid-sporulation (Figure 3B). In agreement, the module's regulatory proteins are two DNA-binding proteins, Hst1 and Sum1, both required for transcriptional repression of middle sporulation-specific genes during vegetative growth and mitosis ([21], Figure 3A). Taken together, these results associate the module with transcription regulation of middle sporulation.

Hst1 and Sum1 are two subunits [1] of the Sum1p/Rfm1p/Hst1p tertiary repression complex controlling middle sporulation genes. RFM1 is a specificity factor that directs the Hst1p histone deacetylase to some of the promoters regulated by Sum1p [22]. Notably, Rfm1 lies in the modules' linkage interval; in fact, it is located within the peak of the interval (Figure 3C). It has an average eQTL likelihood score of 2.6 to its targets, and explains 27% of their gene expression variation. Segregants that inherited the linked locus from the wild RM showed higher expression of the sporulation module's targets than did segregants carrying the locus from the BY strain (Figure 3D). The BY parent carries two polymorphisms at the *RFM1* locus: P247S and N227D. Sequence alignment of six yeast species [23]–[24] showed that the proline residue at position 247 is conserved whereas only the BY strain carries the P247S polymorphism; aspartic acid at position 227 is not evolutionarily conserved (data not shown). This observation suggests that the Ser247 impairs Rfm1 function, perhaps affecting the activity of the entire Sum1/Rfm1/Hst1 complex, leading to residual de-repression of mid-sporulation genes during vegetative growth. The linkage of *RFM1* to expression variation has not been previously shown, probably since the signal could not be detected robustly for a small number of target genes. Our methodology overcomes this problem by exploiting the joint repressive effect of Hst1 and Sum1 during vegetative growth, enabling prediction of the genetic cause of variation in mid-sporulation genes.

**Figure 4:**
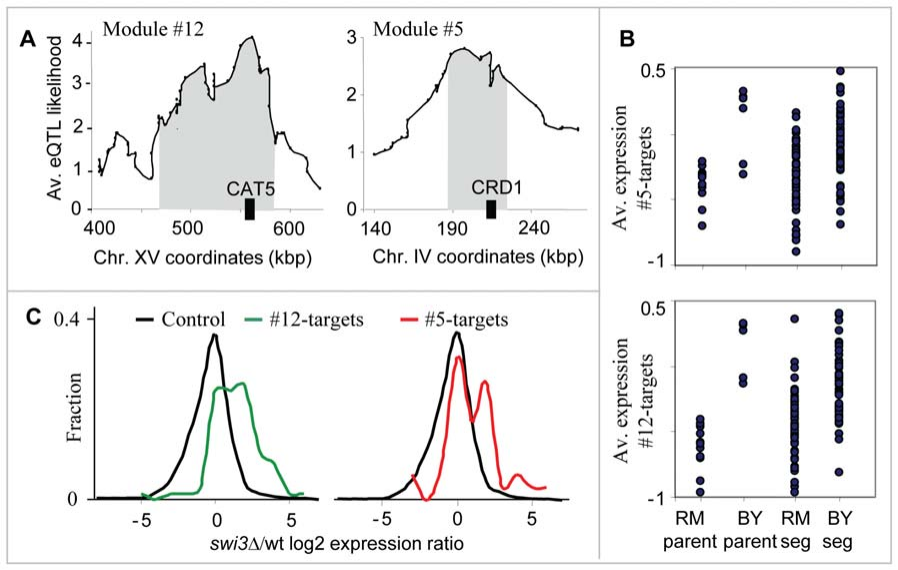
The respiration modules (#12 and #5). (A) The linkage plot. The eQTL likelihood (y-axis) is plotted at each genetic marker (x-axis) residing within or near the linkage interval of modules #12 and #5. The modules' linkage intervals are highlighted in gray. The putative causal regulators *CAT5* and *CRD1* (black boxes), which play a role in respiration, are located within the eQTL likelihood peaks. (B) Expression levels of parents and segregants for a marker in *CCT4* and *RGA1*, which lie near *CRD1* (top) and *CAT5* (bottom). The plots are shown as in Figure 3D. In both cases, the BY parents and segregants carrying the BY allele have higher expression levels than the RM parents and segregants. (C) Distribution of expression values for the target genes of modules #12 (red) and #5 (green), compared with the rest of the genes (black) in *swi3* knockout experiment. Shown are log2-transformed *swi3* vs. wild-type expression ratios [16].

The two respiration modules (#5 and #12) show the ability of our method to identify two distinct linkage intervals sharing the same target genes. The target genes of both modules are enriched with oxidative phosphorylation (P
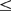
10^−16^ in #5, P
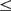
10^−41^ in #12), and generation of precursor metabolites and energy (P
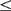
10^−13^ in #5, P
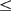
10^−29^ in #12), both of which are related to the process of aerobic cellular respiration, generating energy in the form of ATP (Table S3). The predicted causal regulators are *CRD1* and *CAT5* (modules #5 and #12, respectively), both required for normal respiration functionality and both residing within the peaks of the linkage interval on chromosomes IV and XV, respectively (Figure 4A; only *CAT5* was previously proposed as a causal regulator [6]). Cat5 and Crd1 have an average eQTL likelihood score of 3.5 and 2.6 to their targets, respectively. Cat5 is required for biosynthesis of ubiquinone, an electron-carrying coenzyme in the electron transport chain. Cardiolipin is a phospholipid of the mitochondrial inner membrane, synthesized by the Crd1 cardiolipin synthase. Absence of cardiolipin in *crd1* mutants results in decreased mitochondrial membrane potential and reduced respiration activity [25]. The target genes of the two modules show lower expression in segregants carrying the linked locus from the RM strain compared to the BY strain (Figure 4B).

Our results point to Swi3, but not to the common regulators of respiratory gene expression, as the key mediator of the *CAT5-CRD1* effect. Swi3 is the sole predicted regulator of both respiratory modules (Table S1 and Table 1). Figure 4C demonstrates that indeed, the two respiratory modules are significantly over-expressed in the *swi3* strain (t-test P
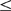
10^−8^ and P
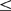
10^−22^ in modules #5 and #12, respectively). Interestingly, the effect of *swi3* deletion is stronger than the deletion effect of known respiratory transcriptional regulators, including Hap2/3/4/5, Mot3, Rox1, Aft1/2, and Cth1/2 (Figure S1). Swi3 is a subunit of the SWI/SNF chromatin remodeling complex, which is required for transcription of a diverse set of genes (e.g., mating-type switching and Gcn4 targets), but its specific role in respiratory gene expression has not been documented.

**Figure 5:**
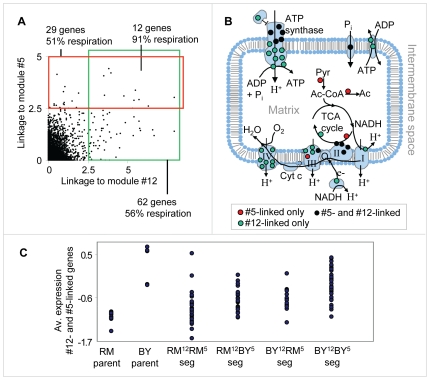
Genetic interactions between the respiration modules. (A) A scatter plot showing the linkage (eQTL likelihood score) to *CAT5*, module #12's putative causal regulator (x axis), and to *CRD1*, module #5's putative causal regulator (y axis), across all genes. Genes that are tightly linked (eQTL likelihood >2.5) to one of the causal regulators are indicated by the red and green boxes. (B) An illustration of mitochondrial respiratory-related reactions and complexes. Shown are the electron transport chain (complexes I, II, III, and IV), the ATP synthase complex, phosphate and ATP/ADP carriers, and the TCA cycle. Proteins that are part of these complexes, whose gene expression is also linked to the putative causal regulators of module #5 only, #12 only, or both, are denoted by red, green and black dots, respectively. (C) Expression levels of parents and segregants for every combination of module #5 and module #12 putative causal regulator alleles. RM^12^-RM^5^ and BY^12^-BY^5^ indicates that both alleles are from the RM and BY parents, respectively. RM^12^-BY^5^ (BY^12^-RM^5^, respectively) indicates that only module #12's allele is from the RM (BY, respectively) strain, whereas module #5's allele is from the other parental strain. The expression levels are averaged over the twelve overlapping target genes depicted in (A). The plot indicates an additive effect of module #5's and module #12's linkage intervals. In plots (A–C), we used the genetic markers residing within *CCT4* and *RGA1*, which are located proximal to the *CRD1* and *CAT5* genes, respectively.

We next investigated the interrelations between the genetic variation in *CAT5* and *CRD1*. To that end, we analyzed all genes that have high linkage (eQTL likelihood >2.5) to either *CAT5* or *CRD1*. Interestingly, the linked genes have a strong overlap: out of the 62 genes linked to *CAT5* and 29 genes linked to *CRD1*, twelve genes are linked to both regulators (hyper-geometric test P
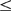
10^−17^) and contain mainly respiratory-related genes (11 of 12, Figure 5A and Table S4). Many of the linked genes are subunits of four respiration-related reactions: the electron transport chain, the citric acid cycle, ATP synthase, and mitochondrial carriers (in total, 15 of 29 in module #5 and 35 of 62 in module #12). Interestingly, the linked genes encode proteins that are non-randomly distributed across the various respiratory complexes: cytochrome c oxidase (Complex IV of electron transport chain) is exclusively encoded by genes linked to *CAT5*; the TCA cycle is composed of proteins encoded by the *CRD1* linked group; and the genes encoding the ATP synthase complex and succinate dehydrogenase (Complex II of electron transport chain) are linked to both *CAT5* and *CRD1* (Figure 5B).

To test for possible genetic interactions, we compared the expression of the twelve overlapping linked genes in segregants carrying four possible combinations of the *CRD1* and *CAT5* alleles. Interestingly, we observed an additive effect of the *CAT5-CRD1* genotypes (Figure 5C; compare also with Figure 4B). Whereas *CAT5* and *CRD1* alone explain 22% and 17% of gene expression variation, respectively, the combination of the two eQTLs *CAT5-CRD1* explains 32% of the gene expression variation. Therefore, our results indicate that a genetic interaction between the eQTL pair *CAT5* and *CRD1* underlies the inheritance of genes required for normal respiration.

## Discussion

Our approach provides a high-resolution tool for identifying functional DNA polymorphisms that affect gene expression. Importantly, it also provides insights into the mechanisms by which genotypes underlie expression changes.

In our method, the regulatory signatures are gene expression profiles that were measured in rich medium under standard conditions on yeast cells carrying a single perturbation. The same methodology can be expanded to handle additional regulatory signature resources. For example, gene expression data measured under a variety of conditions may be included, disclosing modules that are inactive under standard conditions but active under particular extracellular stimuli. Furthermore, protein-DNA binding data, and data from double mutants might provide additional powerful information on ReL modules.

The ReL modules should be interpreted with caution. Genetic linkage does not necessarily imply causality. Two of the three criteria used for identifying the causal regulator are aimed to select among plausible hypotheses but do not demonstrate causality (see Text S2 for details). Additionally, the linkage interval might contain more than one causal polymorphism, whereas ReL analysis assumes a single causal regulator. In the case of two causal polymorphisms located at the same genomic region, ReL analysis might unify them into the same module or fail to detect one of them. Another point to consider is that the ReL modules do not provide an unbiased view of genome-wide genetic linkage. Since the modules are detected based on co-regulation in at least one regulatory signature, the resulting modules depend on the particular signatures included in the compendium. Further, some regulatory relations might be specific to a single regulatory signature, a short linkage interval, or a small number of target genes. ReL analysis may not have enough statistical power to generalize those focused relations into a module. Finally, our modules currently contain only a single linkage interval. Hence, ReL analysis might fail to detect the prevalent case where the target genes are influenced by a combination of multiple interacting loci. It might be possible to extend our framework to detect such interactions automatically. For all these reasons, our method may fail to identify certain correct modules despite a detectable causal polymorphism.

ReL analysis is likely to succeed in organisms other than yeast, including mouse and human. Several genotypic and gene expression datasets are available for these populations [26]–[27], and thus the most prominent obstacle is the lack of a large compendium of mammalian regulatory signatures. Such a resource, however, is likely to be compiled in the future, and the ReL methodology provides a good example of its usefulness. Text S4 provides a quantitative estimation of the number of regulatory signatures required for significant ReL analysis, highlighting the importance of a large compendium. As new technologies for cost-effective count of transcripts in perturbed cells become available (e.g., nCounter [28], shRNA-perturbation), it will be soon easier to obtain a large collection of mammalian regulatory signatures and apply our methodology to them.

When applied to the yeast system, our methodology reveals two intriguing ReL modules. First, we find that DNA polymorphism in *RFM1* underlies gene expression variation of middle sporulation genes. Second, we show that both *CRD1* and *CAT5* underlie gene expression variation in aerobic cellular respiration genes. Further analysis reveals a novel genetic interaction (epistasis) between these two loci. It would be of great interest to explore whether the regulatory mechanisms uncovered here are conserved in other fungal genomes. The discovery here of previously uncharacterized modules and interactions in the well-studied segregating yeast population underscores the importance of large-scale integrated methods in genetic analysis.

## Methods

### Data preparation

We calculated the linkage (an eQTL likelihood score) of genotypic and expression data measured for 112 individuals in a yeast segregating population, as described previously [1]. The linkage matrix represents genetic markers versus genes, where each entry corresponds to the eQTL likelihood score between a given genetic marker and the expression of a given gene. The analysis was applied to all 2956 markers that were genotyped, and all 6230 genes whose gene expression was measured across the segregating population.

We formed a compendium of 283 high-throughput expression profiles obtained from strains mutated in various regulatory proteins [15]–[16]. The compendium includes only strains mutated in a single gene, and each mutant strain is represented by exactly one expression profile. The expression profiles are referred to as regulatory signatures.

### ReL test

Given a genetic marker and a regulatory signature, we evaluate whether genes that are tightly linked to the genetic marker are also over- or under-expressed in the regulatory signature. To that end, we partition the genes into two subsets: genes with high linkage to the genetic marker (denoted high-linkage genes), and the rest of the genes. The difference in the distribution of the regulatory signature values between the two subsets is evaluated using a t-test. The ReL score is the −log_10_ P-value of this t-test (all reported ReL scores are Bonferroni corrected). In our analysis, 11,166 of the 836,548 ReL scores (1.3%) were significant at P<0.001 (see Text S1). Given that the high-linkage (the rest) genes tend to have high (respectively low) regulatory signature values, the group of hit genes includes all those high-linkage genes whose values are above (respectively below) the average regulatory signature value. The hit genes are later used to calculate the target genes of the ReL modules.

The eQTL likelihood threshold, which distinguishes the high-linkage genes from the rest of the genes, was identified as follows: First, genes that are over-expressed and genes that are under-expressed in the regulatory signature are identified. For every possible eQTL likelihood threshold, we test for the over-representation of high-linkage genes in one of these expression groups using a hyper-geometric score (we consider all observed eQTL likelihood values as thresholds). The best score determines the eQTL likelihood threshold. The combination of hyper geometric score and the t-test is important for a robust evaluation. Unlike a t-test, the hyper-geometric test takes into account the amount of high-linkage genes, making sure that the eQTL likelihood threshold is not too high; on the other hand, unlike the hyper-geometric test, the t-test estimates the significance of difference between two distributions. Text S5 demonsrates the robustness of ReL analysis to small changes in the eQTL likelihood threshold.

### Biclustering analysis and ReL modules

The ReL matrix summarizes the ReL scores across all genetic markers and regulatory signatures. We set out to construct a group of co-regulated genes whose common transcription regulation involves both regulatory proteins and a causal regulator. In the ReL matrix, such an event appears as a sub-matrix with significant over-representation of high ReL scores. To identify those sub-matrices, the ISA biclustering algorithm [17] was adapted to work on the ReL matrix. ISA looks for any subset of columns and any subset of rows whose sub-matrix has high scores; the sub-matrix is subject to iterative improvements by adding or removing any column or row. Here we seek sub-matrices with a single range of consecutive genetic markers rather than any subset of markers. To that end, we modified the original ISA so that only markers at the boundaries of the current genetic marker range can be added or removed. On each ISA step, the genetic marker range is optimized efficiently using a dynamic programming algorithm. We start from all possible single entries as seed sub-matrices, and optimize each such seed independently of all others (see Text S6 for details). The resulting sub-matrix is called a ReL module. The ReL score of a module is the average ReL score of its entries.

A ReL module specifies a single range of genetic markers (referred as a linkage interval) and a set of regulatory signatures. For each ReL module, we further compiled the following information:

(i) Each module is associated with a set of regulatory proteins corresponding to the deletion mutants in the module's regulatory signatures. The ReL score of a regulatory protein is its average ReL score over the linkage interval.

(ii) As defined above, each entry of the ReL matrix is associated with a set of hit genes. The module's target genes are all hit genes included in at least 60% of the sub-matrix entries. Here we aim to investigate trans-acting regulation, and therefore, to avoid biases related to cis-acting regulation, genes residing within the linkage interval or less than 30 genes away from it were excluded from the set of target genes. In all thirteen modules under analysis, the original fraction of cis-linked genes was relatively small (Table S2).

Next, the function of the set of target genes is characterized by a hyper-geometric enrichment test using the GO biological process annotation (computed using the EXPANDER software [29]; all reported P-values are corrected for multiple testing). Given one or more significantly enriched biological processes for the same set of target genes, the best scoring process is termed the primary biological process of the module.

(iii) A causal regulator is a gene carrying a polymorphism in its promoter or coding region, which has a trans-acting effect on expression variation of other genes. For each ReL module, we aim to find one or a few putative causal regulators – genes contained within the linkage interval that are highly likely to be the causal regulators of the target genes. Following Tu et al. [7], we predict a putative causal regulator based on the following rules: The causal regulator either plays a role in the primary biological process of the module, or the yeast protein-protein and protein-DNA interaction network contains at least one direct link between the causal regulator and the module's regulatory proteins. Alternatively, the module shows statistical significant enrichment for targets of the causal regulator (see Text S2 for details).

Taken together, a full description of a module includes a set of regulatory proteins, a (small) set of putative causal regulators, and a set of target genes characterized by a primary biological process.

### Additional information

A program implementing our framework is available on the website: http://acgt.cs.tau.ac.il/ReL/.

## Supporting Information

Figure S1 The effect of deletion in respiration transcription regulators on expression in modules #5 and #12. Given a ReL module and a gene expression profile, we applied a t-test to compare the distribution of gene expression values for the module's target genes to the distribution of gene expression values for the rest of the genes. The histogram shows the results of this t-test for profiles taken from strains mutated in respiratory transcription regulators (x axis), using the target genes of module #5 (white) or #12 (black). Y axis: −log P-value of the t-test. Among all respiratory transcription regulators, Swi3 has the strongest effect on the target genes of modules #5 and #12.
(0.07 MB TIF)

Table S1 A full report of all identified modules.
(0.48 MB XLS)

Table S2 Fraction of cis-genes and telomere genes in the ReL modules.
(0.01 MB PDF)

Table S3 Biological processes associated with the ReL modules.
(0.02 MB PDF)

Table S4 CAT5 and CRD1 targets.
(0.03 MB PDF)

Text S1 Evaluating the quality of the results using several statistical models.
(0.03 MB PDF)

Text S2 Identification of putative causal regulator.
(0.04 MB PDF)

Text S3 Evaluation of the learned regulatory proteins.
(0.04 MB PDF)

Text S4 The effect of the compendium size on the ReL analysis.
(0.03 MB PDF)

Text S5 Sensitivity to eQTL likelihood threshold.
(0.02 MB PDF)

Text S6 The biclustering algorithm.
(0.04 MB PDF)
