# Supplementary figures and images for: Understanding Gene Sequence Variation in the Context of Transcription Regulation in Yeast
Source: PLoS Genet. 2010 Jan 8;6(1):e1000800. doi: 10.1371/journal.pgen.1000800 (PMC2794365; doi:10.1371/journal.pgen.1000800)

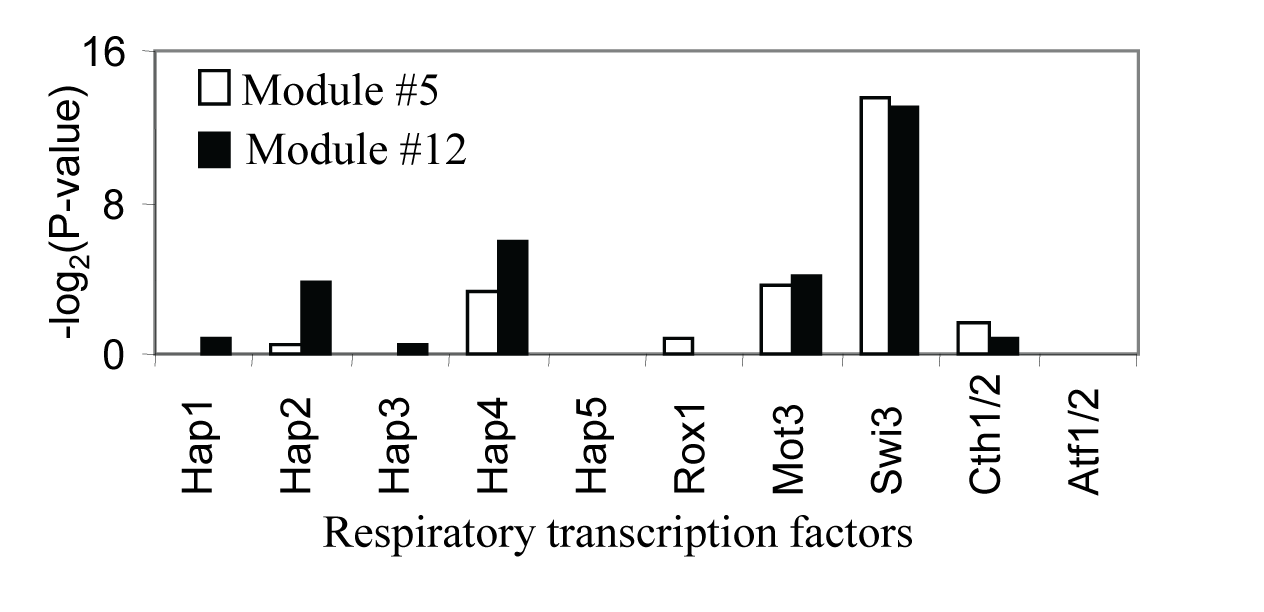

Supplement: Figure S1 — The effect of deletion in respiration transcription regulators on expression in modules #5 and #12. Given a ReL module and a gene expression profile, we applied a t-test to compare the distribution of gene expression values for the module's target genes to the distribution of gene expression values for the rest of the genes. The histogram shows the results of this t-test for profiles taken from strains mutated in respiratory transcription regulators (x axis), using the target genes of module #5 (white) or #12 (black). Y axis: −log P-value of the t-test. Among all respiratory transcription regulators, Swi3 has the strongest effect on the target genes of modules #5 and #12. (0.07 MB TIF) [file pgen.1000800.s001.tif]
