# Supplementary material for: Understanding Gene Sequence Variation in the Context of Transcription Regulation in Yeast
Source: PLoS Genet. 2010 Jan 8;6(1):e1000800. doi: 10.1371/journal.pgen.1000800 (PMC2794365; doi:10.1371/journal.pgen.1000800)
Supplement: Table S2 — Fraction of cis-genes and telomere genes in the ReL modules. (0.01 MB PDF) [file pgen.1000800.s003.pdf]

module ID	linkage interval	ReL score	# target genes	# cis-genes	**Frac. of cis-genes** [#cis-genes /(#cis-genes+#target-genes)]	**Frac. of telomere genes** [#telomere genes / #target genes]	comment
1	II:352-376	3.5	32	4	0.11	0.00	
2	II:489-697	5.5	13	4	0.24	0.06	
3	III:75-105	10.4	12	1	0.08	0.00	
4	III:175-210	11.4	23	2	0.08	0.04	
5	IV:188-226	3.2	28	4	0.10	0.03	
6	V:73-166	11.6	16	3	0.16	0.00	
7	VII:55-73	3.6	29	0	0.00	0.03	
8	VIII:56-140	18.4	18	1	0.05	0.00	
9	X:307-387	6.3	10	2	0.17	0.25	
10	XII:607-748	4.0	27	1	0.04	0.04	
11	XV:170-193	6.8	41	1	0.02	0.02	
12	XV:469-581	4.9	35	7	0.20	0.00	
13	XV:779-880	6.4	17	1	0.06	0.06	
14	IV:1495-1525	9.4	26	4	0.13	0.93	telomere modules - excluded from the analysis
15	X:22-34	7.6	38	4	0.10	0.90	
16	XI:298-315	5.3	25	3	0.11	0.61	
17	XII:782-829	7.5	21	4	0.16	0.76	
18	XII:1014-1067	11.5	30	7	0.19	0.92	

Table S2: Fraction of cis-genes and telomere genes in the ReL modules.
