# Supplementary material for: Understanding Gene Sequence Variation in the Context of Transcription Regulation in Yeast
Source: PLoS Genet. 2010 Jan 8;6(1):e1000800. doi: 10.1371/journal.pgen.1000800 (PMC2794365; doi:10.1371/journal.pgen.1000800)
Supplement: Table S3 — Biological processes associated with the ReL modules. (0.02 MB PDF) [file pgen.1000800.s004.pdf]

module ID	Gene annotation[(a)]	Raw p-value[(b)]	Corrected p-value[(c)]	# target genes	Frequency of annotation
1	ribosome biogenesis - GO0007046	1.8E-07	8.8E-05	32	0.31
2	cytokinesis - GO0000910	4.2E-06	2.1E-03	13	0.38
3	branched chain family amino acid biosynthesis - GO0009082	9.5E-12	4.8E-09	12	0.42
4	response to pheromone during conjugation with cellular fusion - GO0000749	2.4E-11	1.2E-08	23	0.35
5	oxidative phosphorylation - GO0006119	1.4E-18	7.1E-16	28	0.43
	generation of precursor metabolites and energy - GO0006091	1.8E-15	9.0E-13		0.57
	aerobic respiration - GO0009060	1.6E-13	8.0E-11		0.39
	acetyl-CoA metabolism - GO0006084	5.8E-12	2.9E-09		0.25
	coenzyme metabolism - GO0006732	8.4E-12	4.2E-09		0.39
6	pyruvate metabolism - GO0006090	6.0E-06	3.0E-03	16	0.25
	nucleobase biosynthesis - GO:0046122	4.6E-05	2.3E-02		0.18
7	ribosome biogenesis - GO0007046	2.2E-09	1.1E-06	29	0.38
8	conjugation - GO0000746	6.9E-17	3.4E-14	18	0.67
9	zinc-dependent[(d)]			10	0.6
10	ergosterol metabolism - GO0008204	1.3E-20	6.3E-18	27	0.41
	sterol metabolism - GO0016125	1.3E-20	6.6E-18		0.44
11	energy reserve metabolism - GO0006112	4.4E-06	2.2E-03	41	0.12
	carbohydrate metabolism - GO0005975	5.2E-06	2.6E-03		0.22
12	oxidative phosphorylation - GO0006119	3.2E-44	1.6E-41	35	0.66
	generation of precursor metabolites and energy - GO0006091	4.9E-32	2.4E-29		0.74
	cofactor metabolism - GO0051186	2.0E-17	1.0E-14		0.46
	ion transport - GO0006811	2.1E-14	1.0E-11		0.37
13	meiosis-specific or sporulation-specific gene[(d)]			17	0.5

Table S3: Biological processes associated with the ReL modules. (a) GO bioloical process annotations, except for modules 9,13. (b) hyper-geometric test. (c) Bonferromi corrected P-values. (d) For module 9,13, we specify the frequency of the term 'zinc-dependent' , 'meiosis-specific' and 'sporulation-specific' in the description field of the SGD database. For these modules, we provide only the fraction of target genes with this term and omit hyper-geometric P-values.
