# Supplementary material for: Understanding Gene Sequence Variation in the Context of Transcription Regulation in Yeast
Source: PLoS Genet. 2010 Jan 8;6(1):e1000800. doi: 10.1371/journal.pgen.1000800 (PMC2794365; doi:10.1371/journal.pgen.1000800)
Supplement: Table S4 — CAT5 and CRD1 targets. (0.03 MB PDF) [file pgen.1000800.s005.pdf]

Table S4

CAT5 targets				
ORF	Symbol	CAT5-linkage (eQTL likelihood)	Description	Category
YDR322C-A	TIM11	3.6	ATP synthase*	respiration
YPL078C	ATP4	5.7	ATP synthase*	respiration
YLR295C	ATP14	3.9	ATP synthase*	respiration
YPL271W	ATP15	3.9	ATP synthase*	respiration
YDL004W	ATP16	3.4	ATP synthase*	respiration
YDR377W	ATP17	2.6	ATP synthase*	respiration
YML081C-A	ATP18	4.9	ATP synthase*	respiration
YPR020W	ATP20	5.8	ATP synthase*	respiration
YDL181W	INH1	3.2	inhibits ATP hydrolysis by the ATP synthase*	respiration
YGL187C	COX4	3.5	cytochrome c oxidase (Complex IV of electron transport chain)*	respiration
YNL052W	COX5A	2.7	cytochrome c oxidase (Complex IV of electron transport chain)*	respiration
YHR051W	COX6	5.4	cytochrome c oxidase (Complex IV of electron transport chain)*	respiration
YMR256C	COX7	3.0	cytochrome c oxidase (Complex IV of electron transport chain)*	respiration
YLR395C	COX8	2.7	cytochrome c oxidase (Complex IV of electron transport chain)*	respiration
YDL067C	COX9	4.5	cytochrome c oxidase (Complex IV of electron transport chain)*	respiration
YGL191W	COX13	3.4	cytochrome c oxidase (Complex IV of electron transport chain)*	respiration
YMR145C	NDE1	3.1	Mitochondrial external NADH dehydrogenase, provided cytosolic NADH to the mitochondrial respiratory chain*	respiration
YBR085W	AAC3	2.9	Mitochondrial inner membrane ADP/ATP translocase*	respiration
YMR056C	AAC1	3.3	Mitochondrial inner membrane ADP/ATP translocase*	respiration
YML120C	NDI1	3.4	NADH:ubiquinone oxidoreductase (Complex I of electron transport chain)*	respiration
YDR148C	KGD2	3.9	TCA cycle*	respiration
YPR191W	QCR2	3.7	ubiquinol cytochrome-c reductase (Complex III of electron transport chain)*	respiration
YJL166W	QCR8	5.4	ubiquinol cytochrome-c reductase (Complex III of electron transport chain)*	respiration
YGR183C	QCR9	4.3	ubiquinol cytochrome-c reductase (Complex III of electron transport chain)*	respiration
YLR304C	ACO1	3.3	Aconitase, required for the TCA cycle and mitochondrial genome maintenance	mitochondria
YGR046W	TAM41	2.9	Mitochondrial protein involved in protein import into the mitochondrial matrix	mitochondria
YKL093W	MBR1	2.5	Protein involved in mitochondrial functions and stress response; overexpression suppresses growth defects of hap2, hap3, and hap4 mutants	mitochondria
YJR144W	MGM101	3.8	Protein involved in mitochondrial genome maintenance	mitochondria
YJR120W		6.6	Protein of unknown function; mutation causes decreased expression of ATP2 and impaired respiration	mitochondria
YLR168C		6.7	Putative protein of unknown function that may be involved in intramitochondrial sorting	mitochondria
YFR011C		2.8	Putative protein, detected in highly purified mitochondria	mitochondria
YJL103C		2.7	Putative zinc cluster, probably involved in the regulation of energy metabolism	mitochondria
YJL102W	MEF2	4.5	Mitochondrial elongation factor involved in translational elongation	mitochondria
YMR002W	MIC17	3.5	Mitochondrial intermembrane space cysteine motif protein	mitochondria
YBR230C	OM14	3.0	Integral mitochondrial outer membrane protein	mitochondria

CAT5 targets (cont.)				
ORF	Symbol	CAT5-linkage (eQTL likelihood)	Description	Category
YPL075W	GCR1	8.0	Transcriptional activator of genes involved in glycolysis	glycolysis
YJL052W	TDH1	2.7	Protein involved in glycolysis and gluconeogenesis	glycolysis
YCR012W	PGK1	4.3	key enzyme in glycolysis and gluconeogenesis	glycolysis
YKL152C	GPM1	2.7	key enzyme in glycolysis and gluconeogenesis	glycolysis
YEL071W	DLD3	2.6	pyruvate metabolism (cytoplasm)	other
YLL012W	YEH1	3.4	sterol homeostasis	other
YOR009W	TIR4	2.6	Cell wall mannoprotein;required for anaerobic growth	other
YDR384C	ATO3	2.6	Plasma membrane protein	other
YHR033W		2.8	unknown function	other
YMR280C	CAT8	3.6	Zinc cluster transcriptional activator; active after diauxic shift	other
YIL040W	APQ12	3.2	Protein involved in nucleocytoplasmic transport of mRNA	other
YLR224W		3.6		other
YML002W		5.8	unknown function	other
YJL137C	GLG2	3.7	Self-glucosylating initiator of glycogen synthesis	other
YMR226C		2.6	NADP(+)-dependent dehydrogenase	other

CRD1 targets				
ORF	Symbol	CRD1-linkage (eQTL likelihood)	Description	Category
YHR001W-A	QCR10	3.2	ubiquinol cytochrome-c reductase (Complex III of electron transport chain)*	respiration
YBL015W	ACH1	2.5	Acetyl-coA hydrolase, primarily localized to mitochondria*	respiration
YPL262W	FUM1	2.7	TCA cycle*	respiration
YFL018C	LPD1	2.8	component of the pyruvate dehydrogenase and 2-oxoglutarate dehydrogenase multi-enzyme complexes*	respiration
YBL064C	PRX1	2.7	Mitochondrial peroxiredoxin	mitochondria
YER004W		2.7	Protein of unknown function, localized to the mitochondrial outer membrane	mitochondria
YER014W	HEM14	2.5	Mitochondrial enzyme catalyzing the heme biosynthetic pathway	mitochondria
YOL129W	VPS68	2.5	Vacuolar membrane protein of unknown function involved in vacuolar protein sorting; also detected in the mitochondria	other
YPR107C	YTH1	2.8	Essential RNA-binding component of cleavage and polyadenylation factor	other
YHR175W	CTR2	3.6	Putative low-affinity copper transporter	other
YKL146W	AVT3	4.1	Vacuolar transporter	other
YMR191W	SPG5	2.5	unknown function	other
YML119W		3.3	unknown function	other
YBR151W	APD1	2.5	unknown function	other
YNL206C	RTT106	2.5	Protein with a role in regulation of Ty1 transposition	other
YER151C	UBP3	2.6	Ubiquitin-specific protease	other
YDL089W		2.7	unknown function	other

CAT5 and CRD1 targets				
**ORF**	**Symbol**	**CAT5-, CRD1- linkage**	**Description**	**Category**
YBL099W	ATP1	3.8,4.4	ATP synthase*	respiration
YJR121W	ATP2	5.3,2.5	ATP synthase*	respiration
YBR039W	ATP3	5.4,2.9	ATP synthase*	respiration
YDR298C	ATP5	6.3,2.6	ATP synthase*	respiration
YKL016C	ATP7	7.9, 3.0	ATP synthase*	respiration
YKL148C	SDH1	2.5,2.5	succinate dehydrogenase (Complex II of electron transport chain)*	respiration
YLL041C	SDH2	4.7, 3.0	succinate dehydrogenase (Complex II of electron transport chain)*	respiration
YKL141W	SDH3	4.0,4.2	succinate dehydrogenase (Complex II of electron transport chain)*	respiration
YDR178W	SDH4	3.5,3.1	succinate dehydrogenase (Complex II of electron transport chain)*	respiration
YBL045C	COR1	5.5,3.5	ubiquinol cytochrome-c reductase (Complex III of electron transport chain)*	respiration
YJR077C	MIR1	5.7,2.9	Mitochondrial phosphate carrier, imports inorganic phosphate into mitochondria*	respiration
YDR345C	HXT3	3.5,4.0	Low affinity glucose transporter*	other

* Genes that are included in Figure 5B
