# Supplementary material for: Understanding Gene Sequence Variation in the Context of Transcription Regulation in Yeast
Source: PLoS Genet. 2010 Jan 8;6(1):e1000800. doi: 10.1371/journal.pgen.1000800 (PMC2794365; doi:10.1371/journal.pgen.1000800)
Supplement: Text S1 — Evaluating the quality of the results using several statistical models. (0.03 MB PDF) [file pgen.1000800.s006.pdf]

Text S1 - Evaluating the quality of the results using several statistical models.

To evaluate the performance of our algorithm, we compared our results (a single *original ReL matrix* and thirteen *original ReL modules;* **Table 1**) to the results obtained on randomized datasets. Three randomization approaches were tested:

R1 - Reshuffling the names of the genes in the compendium of regulatory signatures. No other reshuffling or randomization was made. This way, we only disrupt the matching between genes in the linkage matrix versus the genes in the compendium. Importantly, the reshuffling maintains all internal relations within the input matrices

R2 – A random permutation of the entire input compendium of regulatory signatures. This way, we completely disrupt the relations within and between the input matrices.

R3 – A full reshuffling of the ReL matrix. This way we maintain the same distribution of ReL scores but disrupt the internal structure of the ReL matrix.

Ten randomized datasets were generated for each randomization method, providing thirty *random ReL matrices* and a total of 78, 4 and 308 *random ReL modules* for R1, R2 and R3, respectively (the same ReL score threshold was applied on both the original and the random ReL modules). **Figure SA1** indicates that the ReL scores in both R1 and R2 random ReL matrices are significantly smaller than ReL scores in the original ReL matrix (KS-test P-values $<< 10^{-22}$, 10^{-30}, respectively). Based on R1 randomization, 11,166 of the 836,548 ReL scores in the original ReL matrix (1.3%) were significant at $P<0.001$. R3 shuffles the original ReL matrix and thus does not affect the distribution of ReL scores. **Figure SA2** demonstrates the number of entries (i.e., number of genetic markers $\times$ number of regulatory signatures) within the random ReL modules vs. the original ReL modules. The plot clearly shows that the original modules tend to have more entries than the random modules (all KS-test P-values $< 10^{-8}$).

To calculate ReL modules' significance, we use the R1 randomization. 5% of the random R1 ReL modules (four out of 78) had more than ten entries, and no random module has more than 44 entries. Recall that the definition of P-value is the probability of obtaining a result at least as extreme as the one that was actually observed, given that the null hypothesis is true. Assuming that the distribution of the number of entries across the random R1 modules reflects the null hypothesis, the P-value of modules #5,#1,#6,#9,#4 is smaller than 0.05, whereas the P-value of all other original ReL modules is smaller than 0.012 (The P-values generated based on R2 and R3 randomization are even better). These P-values take into consideration only the size of the module (i.e., its number of entries) and ignore the ReL score of the module.

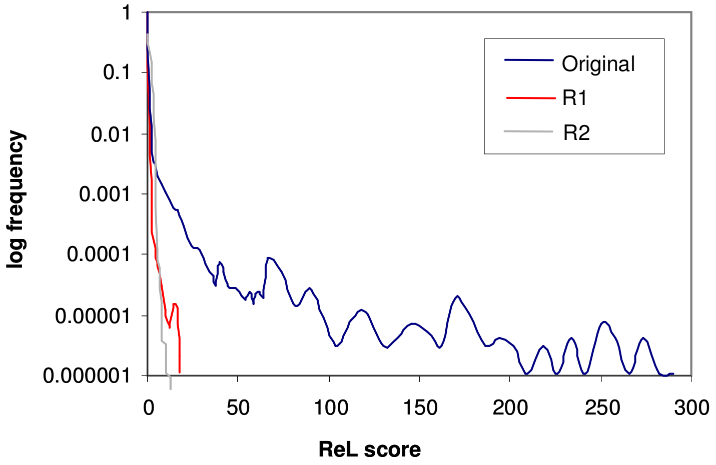

Figure SA1: The distribution of all ReL scores across the original ReL matrix (blue; same as R3) and across R1,R2 random ReL matrices (red, gray).

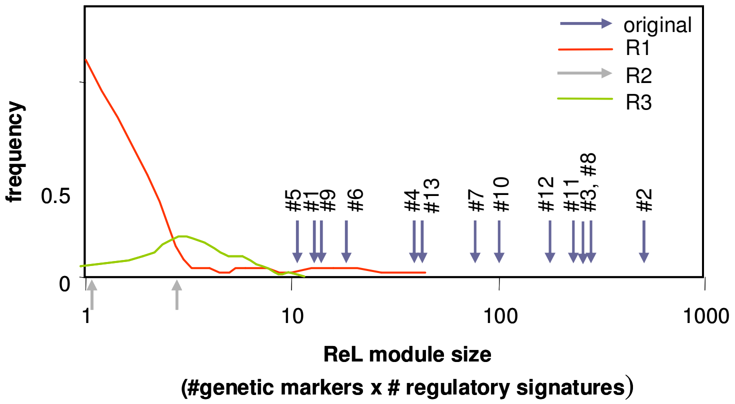

Figure SA2: The distribution of number of entries in random R1 and R3 ReL modules (red, green, respectively). Four random R2 modules (of sizes 1,1,1,3) and the thirteen original ReL modules (accompanied with their module identification number #1-#13) are indicated by gray and blue arrows, respectively.
