# Supplementary material for: Understanding Gene Sequence Variation in the Context of Transcription Regulation in Yeast
Source: PLoS Genet. 2010 Jan 8;6(1):e1000800. doi: 10.1371/journal.pgen.1000800 (PMC2794365; doi:10.1371/journal.pgen.1000800)
Supplement: Text S2 — Identification of putative causal regulator. (0.04 MB PDF) [file pgen.1000800.s007.pdf]

Text S2: Identification of putative causal regulator.

Our approach builds on earlier work of Tu et al. [1]. For each module, we first identified the primary biological process (**Table S3**) and the best-scoring regulatory protein. Next, we identified genes within the linkage interval whose encoded protein have either (i) the same primary biological process as the best-scoring regulatory protein, (ii) a physical interaction with the best-scoring regulatory protein (**Table SB1**), or (iii) the module shows statistically significant enrichment for targets of the causal regulator (a hyper-geometric enrichment test for ChIP-chip data of Harbison et al. [2] using a protein-DNA binding cutoff $P = 10^{-5}$). The two former criteria are aimed to identify plausible hypotheses but do not demonstrate causality. The latter criterion provides causal information but can be applied only for those proteins for which DNA binding data are available.

The putative causal regulator was determined based on evidence from the above three criteria (see **Table SB2**). In particular, the putative causal regulators *TRM7, MAT, CRD1, TAN1* and *IRA2* were determined based on the biological process evidence. The putative causal regulators *AMN1, LEU2, URA3, GPA1* and *RFM1* were determined based on both process and interaction evidence. The putative causal regulators *ZAP1* and *HAP1* were determined based on enrichment for their targets. For module #12, we choose *CAT5* (rather than *CRC1* and *PTC5*) since it appears in the peak of the eQTL likelihood (**Figure 4A**). Among the thirteen putative causal regulators, only *HAP1* and *ZAP1* are supported by causal information (column 6 of **Table SB2**).

Module ID	Protein-protein and protein-DNA interactions
2	ACE2 <-> ICS2
2	ACE2 <-> AMN1
2	ACE2 <-> CDC28
3	LEU3 <-> LEU2
4	STE12 <-> ELO1 <-> FEN1
4	STE12 <-> CHS7 <-> FEN1
4	STE12 <-> AXL1 <-> BUD5
4	STE12 <-> TUP1 <-> MATALPHA2,ALPHA2
4	STE12 <-> MCM1 <-> MATALPHA2,ALPHA2
4	STE12 <-> MCM1 <-> MATALPHA1,ALPHA1
4	STE12 <-> TAF9 <-> TAF2
5	SWI3 <-> SNF11 <-> RPN5
5	SWI3 <-> TAF14 <-> RPO21
6	PPR1 <-> URA3
8	STE12 <-> GPA1
9	TEC1 <-> HAP1 <-> UBX6
9	TEC1 <-> HHF1 <-> RTT101
9	TEC1 <-> MSB2 <-> MAD2
13	HST1 <-> RFM1
13	SUM1 <-> RFM1

Table SB1: Interactions between regulatory proteins and putative causal regulators. We generated a comprehensive *S. cerevisiae* protein-protein and protein-DNA interaction network by combining information from the interaction databases SGD, BioGRID, BIND

and [3]. The table contains only short pathways between the regulatory proteins (red) and causal regulators (blue) within this network (direct interactions or paths of length 2).

Module ID	Primary biological process	Best-scoring regulatory protein	Evidence from biological process	Evidence from interaction network	Evidence from binding to targets	Putative causal regulator
1	ribosome biogenesis	PIB2	TRM7			TRM7
2	cytokinesis	ACE2	AMN1, CSH1	AMN1 , ICS2, CDC28		**AMN1**
3	branched chain aa biosynthesis	LEU3	LEU2	LEU2		**LEU2**
4	response to pheromone	STE12	MATALPHA1,2			**MATALPHA1,2**
5	oxidative phosphorylation	SWI3	CRD1			CRD1
6	pyruvate metabolism	PPR1	URA3	URA3		**URA3**
7	ribosome biogenesis	STB3	TAN1			TAN1
8	conjugation	STE12	GPA1,STE20	GPA1		**GPA1**
9	zinc-dependent	TEC1	ZAP1		ZAP1	ZAP1
10	ergosterol metabolism	REB1			HAP1	**HAP1**
11	energy reserve metabolism	MGA2	IRA2			**IRA2**
12	oxidative phosphorylation	SWI3	CAT5,CRC1, PTC5			CAT5
13	meiosis-specific	SUM1, HST1	RFM1	RFM1		RFM1

Table SB2: Identification of the putative causal regulator. For each module (column 1), the table presents its primary biological process (column 2), its best-scoring regulatory protein (column 3), plausible causal regulators that have the same primary biological process (column 4), a physical interaction with the best-scoring regulatory protein (column 5), or statistically significant enrichment for their binding targets (column 6; FDR-corrected hyper-geometric P-value < 0.01). Based on the collection of evidence in columns 4-6, column 7 provides the predicted 'putative causal regulator'. Previously verified causal regulators are marked in bold. In the case of *ZAP1* and *CAT5*, the causal regulators were predicted only computationally [4,5] but have not been experimentally tested. In module #13, *SUM1* and *HST1* have similar ReL scores (STable 1).

1. Tu Z, Wang L, Arbeitman MN, Chen T, Sun F (2006) An integrative approach for causal gene identification and gene regulatory pathway inference. Bioinformatics 22: e489-496.
2. Harbison CT, Gordon DB, Lee TI, Rinaldi NJ et al. (2004) Transcriptional regulatory code of a eukaryotic genome. Nature 431: 99–104.
3. Guelzim N, Bottani S, Bourgine P, Kepes F (2002) Topological and causal structure of the yeast transcriptional regulatory network. Nat Genet 31: 60-63.
4. Lee SI, Pe'er D, Dudley AM, Church GM, Koller D (2006) Identifying regulatory mechanisms using individual variation reveals key role for chromatin modification. Proc Natl Acad Sci U S A 103: 14062-14067.
5. Yvert G, Brem RB, Whittle J, Akey JM, Foss E, et al. (2003) Trans-acting regulatory variation in Saccharomyces cerevisiae and the role of transcription factors. Nat Genet 35: 57-64.
