# Supplementary material for: Understanding Gene Sequence Variation in the Context of Transcription Regulation in Yeast
Source: PLoS Genet. 2010 Jan 8;6(1):e1000800. doi: 10.1371/journal.pgen.1000800 (PMC2794365; doi:10.1371/journal.pgen.1000800)
Supplement: Text S3 — Evaluation of the learned regulatory proteins. (0.04 MB PDF) [file pgen.1000800.s008.pdf]

Text S3: Evaluation of the learned regulatory proteins in comparison to Geronemo, Zhu et al. and Lirnet.

eQTL hotspot [1,2]	functional enrichment [1,2]	known TF + reference	Geronemo	Zhu et al.	Lirnet	ReL analysis
LEU2	Leucine biosynthesis	Leu3, Gcn4 [3]	#86 - Sip4, Pnc1	Blue - Gcn4		#3 - Leu3
MAT	Mating	Ste12, Dig1,2 [4]		Cyan - Ste12		#4 - Ste12
URA3	Uracil biosynthesis	Ppr1 [5]	#78 - Mih1		#137 - Mth1	#6 - Ppr1
GPA1	Pheromone response	Ste12, Dig1,2 [1,2,4]		Midnight blue - Dig1,2	#99 - no prediction	#8 - Ste12
AMN1	Budding, cytokinesis	Ace2 [2]	#43 - Nrg1,Msb2	Turquoise/green - Fhl1, Reb1		#2 - Ace2
HAP1	Ergosterol metabolism	Hap1 [6]	#76 - Hsl1, Bas1	black - Hap1		#10 - Reb1

We compared our results with those of several recent methods (Geronemo: Lee et al. 2006, Lirnet: Lee et al. 2009, and Zhu et al. 2008), which were applied to the same yeast eQTL dataset (Brem et al. 2002). All compared methods output a set of ‘modules’, each comprising a group of target genes, and zero, one or more causal regulators and regulatory proteins. The analysis here refers only to those modules with at least one causal regulator.

We evaluated how well the compared methods recover the regulatory proteins of the modules. As gold standard modules are not available, we performed the comparison on the six verified eQTL hotspots from Yvert et al. (2003) and Brem et al (2002) (column 1). For each of the six hotspots, the enriched biological process of the linked genes is presented in column 2 (Yvert et al. (2003) and Brem et al (2002)). The known transcription factor of the target genes, based on well-established biological knowledge, is presented in column 3 and accompanied with a literature citation. Next we assigned, whenever possible, modules to eQTL hotspots. An assigned module satisfied the following two criteria: First, the genomic location of the eQTL hotspot must reside within the module’s linkage interval. Second, the enriched process of the module must be the same as the hotspot’s enriched process (eQTL hotspot enrichment is detailed in column 2; modules’ enrichment are as detailed in their original publications: Geronemo - STable 2 and STable 4 in Lee et al. 2006; Lirnet - STable 5 in Lee et al. 2009; Zhu et al. - Table 1 in Zhu et al. 2008; ReL analysis - STable 3 here). The assigned modules from all four compared methods are presented in columns 4-7, together with their predicted regulatory proteins. The modules are labeled as in the original publications. A module’s regulatory protein is considered as ‘correct prediction’ if it is a known regulator of the target genes (i.e., if it appears in column 3)**.** Correct predictions are highlighted in red.

As an example, the linkage interval of Zhu et al.’s ‘Midnight blue’ module contains *GPA1* and the target genes are enriched in pheromone response. Based on this information, ‘Midnight blue’ was assigned to the *GPA1* eQTL hotspot. According to Zhu et al., ‘Midnight blue’ has two predicted regulatory proteins, Dig1 and Dig2. Based on their match to the known regulators of the system in column 3 (Dig1,2 and Ste12), we say that Zhu et al. have predicted correctly the regulatory proteins of the ‘Midnight blue’ module, and highlight this correct prediction in red.

The table indicates the ability of ReL analysis to correctly identify regulatory proteins and compares it with other methods. Out of six well-characterized eQTL hotspots, ReL analysis learns successfully five modules and predicts correctly five regulatory proteins. For comparison, Geronemo, Zhu et al. and Lirnet learn only four, five and two modules, and predict correctly only zero, four and zero regulatory proteins, respectively. Interestingly, the Ppr1 protein was detected only by the ReL analysis.

[1] Yvert G, Brem RB, Whittle J, Akey JM, Foss E, et al. (2003) Trans-acting regulatory variation in Saccharomyces cerevisiae and the role of transcription factors. Nat Genet 35: 57-64 .
[2] Brem RB, Yvert G, Clinton R, Kruglyak L (2002) Genetic dissection of transcriptional regulation in budding yeast. Science 296: 752-755.
[3] Kohlhaw GB (2003) Leucine biosynthesis in fungi: entering metabolism through the back door. Microbiol Mol Biol Rev 67: 1-15.
[4] Roberts CJ, Nelson B, Marton MJ, Stoughton R, Meyer MR, et al. (2000). Signaling and circuitry of multiple MAPK pathways revealed by a matrix of global gene expression profiles. Science 287: 873-880.
[5] Flynn PJ, Reece RJ (1999) Activation of transcription by metabolic intermediates of the pyrimidine biosynthetic pathway. Mol Cell Biol 19: 882-888.
[6] Gaisne M, Becam AM, Verdiere J, Herbert CJ (1999) A 'natural' mutation in Saccharomyces cerevisiae strains derived from S288c affects the complex regulatory gene HAP1 (CYP1). Curr Genet 36: 195-200.120
