# Supplementary material for: Understanding Gene Sequence Variation in the Context of Transcription Regulation in Yeast
Source: PLoS Genet. 2010 Jan 8;6(1):e1000800. doi: 10.1371/journal.pgen.1000800 (PMC2794365; doi:10.1371/journal.pgen.1000800)
Supplement: Text S4 — The effect of the compendium size on the ReL analysis. (0.03 MB PDF) [file pgen.1000800.s009.pdf]

Text S4: The effect of the compendium size on the ReL analysis.

The results of the ReL analysis depend on the particular signatures included in the compendium. Here we aim to assess the relations between the compendium size (number of regulatory signatures) and the number of resulting ReL modules. In particular, we aim to ask when is a compendium too small to expect an interesting result. Answering this question will shed light on the amount of information required for ReL analysis in other organisms.

To estimating the required size of dataset, we apply the ReL analysis on small fractions of the compendium in a bootstrap-like manner. In each test, we re-sample (without replacement) a small fraction of the compendium and apply the ReL analysis only on the resulting partial compendium. We performed ten tests for each of the compendium fractions λ = 0.05, 0.15 and 0.25 (14, 42 and 70 out of 283 regulatory signatures, respectively), producing a collection of *partial ReL modules*. A partial module forms a *hit* when it matches one of the original ReL modules. Three criteria must hold for such a match: The two modules must share at least 30% of the linkage range and 30% of the target genes, and 50% of the regulatory proteins included in one of the modules must be also included in the other. The analysis excludes cis modules and considers only modules with more than ten entries.

Figure SD1 presents the number of hits based on their match to the eighteen original ReL modules reported in **Table S2** (including telomere modules). The analysis indicates the importance of a large compendium: given a few dozen yeast regulatory signatures (λ = 0.15), ReL analysis provides only zero to eight partial ReL modules. With the large number of regulators and complex regulation in mammals, ReL analysis in these organisms requires a compendium of a few hundred signatures.

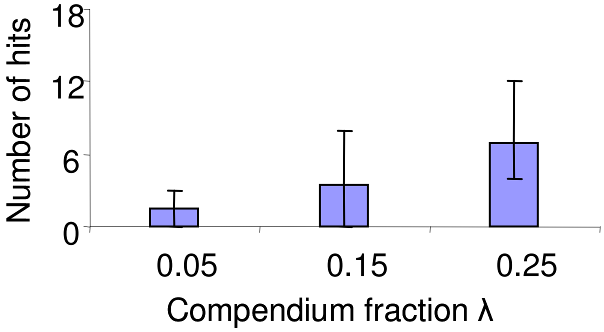

Figure SD1: Number of partial module hits (y axis) obtained using a fraction λ of the compendium (x axis). For each fraction λ, the histogram presents the median (bars), maximum and minimum (error bars) number of hits across the ten repeats.
