# Supplementary material for: Understanding Gene Sequence Variation in the Context of Transcription Regulation in Yeast
Source: PLoS Genet. 2010 Jan 8;6(1):e1000800. doi: 10.1371/journal.pgen.1000800 (PMC2794365; doi:10.1371/journal.pgen.1000800)
Supplement: Text S5 — Sensitivity to eQTL likelihood threshold. (0.02 MB PDF) [file pgen.1000800.s010.pdf]

Text S5: Sensitivity to eQTL likelihood threshold

Given a genetic marker, we partitioned the genes into two subsets: genes with high linkage to the marker and the rest of the genes (see **Methods**). The partition threshold was optimized based on a hyper-geometric test, and therefore called an *optimized threshold*. Here we test the sensitivity of the algorithm to this optimized threshold. We apply two approaches for threshold selection: **Fixed threshold** – the threshold is genome-wide error rate α. Thus, a fraction α of the genes have an eQTL likelihood that is equal or higher than the threshold. We tested six error rates between $\alpha = 0.005$ and $\alpha = 0.03$. **Noisy threshold** – for each genetic marker and regulatory protein, we use threshold $\mu+\varepsilon$ where μ is the optimized threshold and $\varepsilon \sim N(0,\nu)$. We tested four noise levels between $\nu=0.05$ and $\nu=1$, ten repeats of each.

Figure SE1 demonstrates the sensitivity of the results to noisy and fixed threshold. As a sensitivity measure, we use the number of *hits* – the number of original modules that were also detected when using a fixed or noisy threshold (for a formal definition of hits, see **Text S4**). The plots clearly demonstrate that many of the modules are robust to a fixed or noisy threshold.

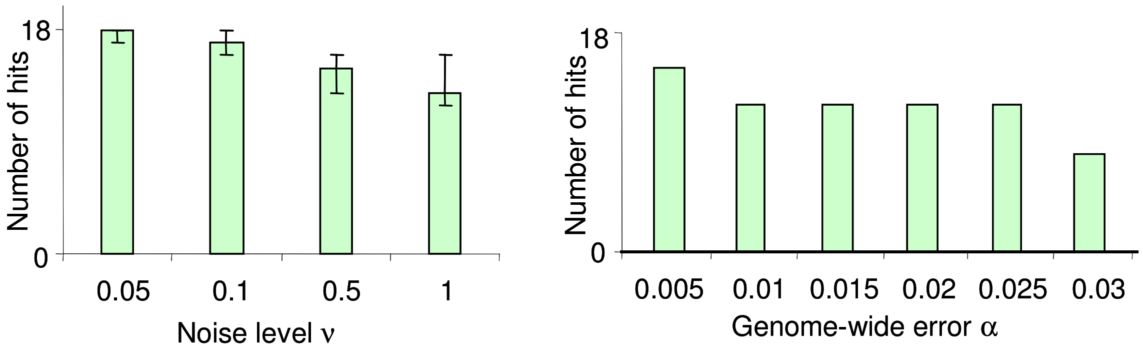

Figure SE1. Quality of the fixed and noisy threshold approach. X axis: noise level ν (left panel) and error rate α (right panel). Y axis: number of hits. The hits were calculated based on matching to the eighteen original ReL modules. The left panel presents median (bars), maximum and minimum (error bars) number of hits across ten repeats.
