# Supplementary material for: Understanding Gene Sequence Variation in the Context of Transcription Regulation in Yeast
Source: PLoS Genet. 2010 Jan 8;6(1):e1000800. doi: 10.1371/journal.pgen.1000800 (PMC2794365; doi:10.1371/journal.pgen.1000800)
Supplement: Text S6 — The biclustering algorithm. (0.04 MB PDF) [file pgen.1000800.s011.pdf]

Text S6: The biclustering analysis

Definitions:

1. A *ReL matrix M*: rows are genetic markers $g_1,\ldots,g_m$ and columns are regulatory signatures $c_1,\ldots,c_n$. Each entry M_{ij} provides the ReL score for genetic marker g_i and signature c_j.
2. A *ReL module* (G,C) is a subset of signatures C and a range of consecutive genetic markers G.
3. The ReL score of a module (G,C) $= \underset{i\in G, j\in C}{average}(M_{i,j})$
4. In biclustering iteration *k*, module *l* is denoted by (G_k^l, C_k^l), where G_k is range of consecutive genetic markers and C_k is the subset of signatures in the *k*-th iteration of module *l*.

Input:

1. A *ReL matrix M*.
2. A *score shift t*: a constant parameter.
3. A *seed threshold s*: a constant parameter.
4. A *redundancy threshold r*: a constant parameter.
5. A *module threshold q*: a constant parameter.

In the current study, we used *t*=2, *s*=4, *r*=0.8 and *q*=3.

Goal

Given *M, t, s, r*, and *q,* compute a set of ReL modules with maximal ReL scores.

Heuristic approach:

Finding the set of *k* ReL-modules with the best ReL scores is computationally intractable (see, e.g., Ihmels et al. 2001; Tanay et al. 2002). Here we apply a heuristic approach called ISA (Ihmels et al. 2001): We start with a set of seed modules. Each seed is improved iteratively by choosing the best set of signatures for a given genetic marker range and then the best range for the given subset of signatures. Note that the standard ISA looks for any subset of columns and any subset of rows that attain high scores. Unlike the standard algorithm, here we focus on sub-matrices with a single range of consecutive genetic markers rather than any subset of markers. To that end, we modified the original ISA approach as follows: In each iteration k, choosing the best genetic marker range is done by starting form the best marker in iteration k-1 and optimizing the range efficiently using a dynamic programming algorithm.

Initialization:

- Create a set of seeds. A *seed* is a ReL module (G_0,ϕ) where the subset of regulatory signatures is empty, G_0 contains a pair of consecutive genetic markers, and for each $i \in G_0$, $\max_{j=1,\ldots,n}\left(M_{i,j}\right) > s$.
- $\forall ij \;\; M'_{i,j} = M_{i,j} - t$; $q' = q - t$

Iterative algorithm:

for each seed (G_0, ϕ)

for $k = 0$ until G_k converges do

// Create the set C_k

for $j = 1$ to n do

if $\sum_{i \in G_k} M'_{i,j} > 0$ add signature j to C_k

// Create the set G_{k+1} by extending the best genetic marker to a range.

$rangeCenter = \arg\max_{i \in G_K} (\underset{j \in C_K}{average}(M'_{i,j}))$

$accumulatedScore_{rangeCenter} = \max_{i \in G_K} (\underset{j \in C_K}{average}(M'_{i,j}))$

// Extend the *rangeCenter* to one side until *rangeEnd*

$l = rangeCenter$

while ($accumulatedScore_l > 0$)

$l{+}{+}$

$accumulatedScore_l = accumulatedScore_{l-1} + \underset{j \in C_k}{average}(M'_{l,j})$

$rangeEnd = \underset{p=rangeCenter,\ldots,l}{\arg\max} \; accumulatedScore_p$

// Extend the range to the other side

$l = rangeCenter$

while ($accumulatedScore_l > 0$)

$l{-}{-}$

$accumulatedScore_l = accumulatedScore_{l+1} + \underset{j \in C_k}{average}(M'_{l,j})$

$rangeStart = \underset{p=l,\ldots,rangeCenter}{\arg\max} \; accumulatedScore_p$

$G_{k+1} = \{rangeStart, \ldots, rangeEnd\}$

Report (G_k, C_k) *iff* $\underset{i \in G_{k+1}, j \in C_k}{average}(M'_{i,j}) > q'$

Filtering overlapping modules

We check the percentage of overlap between the linkage intervals of each two modules. In case two modules overlap by > *r* percent, the module with lower ReL score is removed.

- Ihmels J, Friedlander G, Bergmann S, Sarig O, Ziv Y, et al. (2002) Revealing modular organization in the yeast transcriptional network. Nat Genet 31: 370-377.
- Tanay A, Sharan R, Shamir R. (2002). Discovering statistically significant biclusters in gene expression data. Bioinformatics. 18 S1:S136-44.
